# Pegvisomant in combination or pegvisomant alone after failure of somatostatin analogs in acromegaly patients: an observational French ACROSTUDY cohort study

**DOI:** 10.1007/s12020-020-02501-3

**Published:** 2020-09-28

**Authors:** Emmanuelle Kuhn, Philippe Caron, Brigitte Delemer, Isabelle Raingeard, Hervé Lefebvre, Gérald Raverot, Christine Cortet-Rudelli, Rachel Desailloud, Clementine Geffroy, Robin Henocque, Yves Brault, Thierry Brue, Philippe Chanson

**Affiliations:** 1 Assistance Publique-Hôpitaux de Paris, Hôpital Bicêtre, Centre de Référence des Maladies Rares de l’Hypophyse HYPO, 94275 Le Kremlin-Bicêtre, France; 2 Université Paris-Saclay (Université Paris-Sud), Inserm, Signalisation Hormonale, Physiopathologie Endocrinienne et Métabolique, Le Kremlin-Bicêtre, France; 3 CHU de Toulouse, Hôpital Larrey, 24 Chemin de Pouvourville, TSA 30030, 31059 Toulouse Cedex 9, France; 4 CHU de Reims—Hôpital Robert Debré, Avenue du Général Koenig, 51092 Reims Cedex, France; 5 CHRU de Montpellier, Maladies Endocriniennes, Hopital Lapeyronie, 295 Avenue du Doyen Gaston Giraud, 34295 Montpellier Cedex 5, France; 6 CHU de Rouen, 1 Rue de Germont, 76031 Rouen Cedex, France; 7 Hospices civils de Lyon, Hôpital Louis Pradel, 59 Boulevard Pinel, 69677 Bron Cedex, France; 8 CHR Lille, Hôpital Claude Huriez, Rue Michel Polonovski, 59037 Lille, France; 9 CHU d’Amiens, Hôpital Nord, Place Victor Pauchet, 80054 Amiens Cedex 1, France; 10 Pfizer France, 23-25 Avenue du Docteur Lannelongue, 75668 Paris Cedex 14, France; 11 CHU de Marseille, Hôpital de la Conception, 147 boulevard Baille, 13385 Marseille Cedex 5, France

**Keywords:** Acromegaly, GH receptor antagonist, Somatostatin analogs, Combination therapy

## Abstract

**Objective:**

After surgery, when somatostatin analogs (SAs) do not normalise IGF-I, pegvisomant (PEG) is indicated. Our aim was to define the medical reasons for the treatment of patients with PEG as monotherapy (M) or combined with SA, either as primary bitherapy, PB (PEG is secondarily introduced after SA) or as secondary bitherapy, SB (SAs secondarily introduced after PEG).

**Methods:**

We retrospectively analysed French data from ACROSTUDY.

**Results:**

167, 88 and 57 patients were treated with M, PB or SB, respectively, during a median time of 80, 42 and 70 months. The median PEG dose was respectively 15, 10 and 20 mg. Before PEG, the mean IGF-I level did not differ between M and PB but the proportion of patients with suprasellar tumour extension was higher in PB group (67.5% vs. 44.4%, *P* = 0.022). SB regimen was used preferentially in patients with tumour increase and IGF-I level difficult to normalise under PEG. In both secondary regimens, the decrease of the frequency of PEG’s injections, compared to monotherapy was confirmed. However, the mean weekly dose of PEG between M and PB remained the same.

**Conclusions:**

The medical rationale for continuing SAs rather than switching to PEG alone in patients who do not normalise IGF-I under SAs was a tumour concern with suprasellar extension and tumour shrinkage under SA. A potential explanation for introducing SA in association with PEG appears to be a tumour enlargement and difficulties to normalise IGF-I levels under PEG given alone. In both regimens, the prospect of lowering PEG injection frequency favoured the choice.

## Introduction

Several therapeutic options (surgery, medical treatment and radiotherapy) may be used to manage acromegaly [1–4]. The first-line therapy is surgical removal of the pituitary GH-secreting adenoma, which normalises GH/IGF-I levels in 40–70% of patients [5–8], depending on tumour size (microadenomas are more amenable to cure), preoperative GH concentration (the success rate is higher when GH concentrations are low, i.e., <10 µg/L or 30 mIU/L) and surgeon experience. Pharmacological therapy is generally preferred for second-line treatment for persistent disease after surgery, whereas radiotherapy is usually considered as a third-line therapy option. Three classes of pharmacological treatment are available for acromegaly: somatostatin analogues (SAs), dopamine agonists (DAs) and GH receptor antagonists (pegvisomant, PEG). When SAs have been used as the primary medical treatment, IGF-I levels have normalised in about half of patients [9, 10]. While PEG therapy has allowed almost all patients to achieve IGF-I normalisation in pivotal trials [11, 12], roughly two-thirds of patients normalise IGF-I levels in surveillance and real-life studies [13–16]. Because of their distinct modes of action, it could be useful to combine PEG with SAs when patient conditions are not controlled by SAs alone [2]. In observational studies with patients treated by both drugs, it has been shown that combination treatment is effective in controlling IGF-I level [17–21], and potentially enables a lower dose of PEG, compared with PEG alone [20]. Quality of life was shown to improve in patients treated with the combination therapy, compared to patients treated by SA alone, while the IGF-I levels were similar between both groups [22], although this was not confirmed in two later studies [21, 23]. Whether PEG in combination with SA, in comparison to PEG alone, provides any advantage in terms of dose, side effects and efficacy in real life has not been well documented.

We took advantage of the ACROSTUDY, a long-term observational study that documented real clinical practice in patients with acromegaly treated with PEG, to define the medical reasons that lead physicians to choose to treat a French cohort of patients with either PEG alone or a combination of PEG and SA. We also compared the efficacy, doses and side effects of both regimens (PEG monotherapy (M) or PEG + SA combined therapy). We chose to analyse, along with IGF-I level, the patient characteristics (disease severity, tumour size and extrasellar expansion, tumour shrinkage under SA, abnormal glucose tolerance) and the practical reasons (required doses, tolerance, injection frequency, compliance) that could explain the choice of regimen. We divided the patients from the French clinical practice cohort into three regimen groups: patients treated with PEG alone (M), patients treated with PEG in combination with SA (primary bitherapy (PB)) and patients initially treated with PEG alone and secondarily with PEG in combination with SA (secondary bitherapy (SB)). This analysis was not done in previous ACROSTUDY publications.

## Patients and methods

### Study design

ACROSTUDY is an open-label, global, non-interventional, longitudinal, post-marketing surveillance study that included patients with acromegaly who were treated with PEG. All collected patient data were from routine clinical practice. All patients gave their informed consent for the study. ACROSTUDY included 2221 patients who were treated with PEG from 14 countries, between 2004 and 2018, and provided long-term follow-up for safety and treatment outcomes. In France, the study was not approved by a medical ethical or institutional review board as not deemed necessary by the French law for observational non-interventional studies on humans at the time of initiation. However, formal approval of participants was obtained for using patients data for study purposes and data handling was performed according to national laws. The study was approved by the Comité Consultatif sur le Traitement de l’Information en matière de Recherche dans le domaine de la Santé (Ministère Délégué de l’Enseignement Supérieur et de la Recherche) in 2006 (N°06.83) and by Commission Nationale Informatique et Libertés (N°1178306) in 2007.

The data of the French ACROSTUDY (interim analysis, August 2016) allowed us to identify two frequent PEG + SA combination regimens in French clinical practice. We differentiated M when PEG was used alone, PB when PEG was added to prior SA treatment and SB when SA was added secondarily after PEG M.

The first objective of this descriptive analysis was to define the medical (through patient characteristics) and practical reasons from the physicians leading to the choice of M, PB and SB regimens in the French cohort. The second objective was to compare the efficacy of the three regimens in terms of IGF-I normalisation, dose and tolerance.

To understand and define the treatment choice rationale, we compared M to PB and M to SB. The patient characteristics and treatment data were compared between baseline and after PEG initiation for each treatment regimen. After PEG initiation, the first and the last IGF-I levels of each treatment regimen were analysed as well as the PEG posology at the time where these IGF-I levels were assessed. Baseline was defined as the period before PEG initiation. After PEG initiation, the first and the last IGF-I levels of each treatment regimen were analysed as well as the PEG posology at the time where these IGF-I levels were assessed. For SB, data after PEG treatment and before SA introduction were also considered.

### Inclusion/exclusion criteria

Patients who received at least one dose of PEG and patients on SA in combination with PEG were included. The other inclusion/exclusion criteria were the same as those of ACROSTUDY [13].

### Statistical analysis

The full analysis set included all French patients enroled in ACROSTUDY. Data are reported as mean ± standard deviation or median with first and third quartiles (Q1, Q3) according to the distribution or percentages, as appropriate. IGF-I and GH levels were log-transformed, and results are expressed as geometric means. As the three patient groups were not defined by a randomisation process, between-group comparisons were considered exploratory. Continuous variables were analysed using the Student’s *t* test or Mann–Whitney *U* test for independent samples and the paired *t* test or sign test for paired samples. Chi-square and Fisher’s exact tests were used to compare categorical variables between groups, and a McNemar’s test was used for paired samples. The time needed to achieve first normal IGF-I level was described using Kaplan–Meier estimates and compared using a log-rank test. All tests were two-sided, and *P* values < 0.05 were considered statistically significant. *P* values are nominal and were not adjusted for multiplicity; estimates are provided with 95% confidence intervals; and missing data were not imputed. Statistical analyses were performed using SAS statistical software version 9.4 (SAS Institute, Cary, NC), and the interim analysis was performed in August 2016.

## Results

### Demographic characteristics

We analysed data from 312 patients who were enroled from the French centres of ACROSTUDY. The main demographic characteristics of the patients are given in Table 1. By definition, all patients were being treated with PEG when they were included in this study. The number (%) of patients in the three groups: M, PB and SB cohorts, for this descriptive analysis was: 167 (53.5%), 88 (28.2%) and 57 (18.3%), respectively.

**Table 1:** Baseline patient characteristics and concomitant treatments

	Monotherapy (*n* = 167)	Primary biotherapy (*n* = 88)	Secondary biotherapy (*n* = 57)	Total (*n* = 312)
Sex				
Male	79 (47.3%)	51 (58.0%)	35 (61.4%)	165 (52.9%)
Female	88 (52.7%)	37 (42.0%)	22 (38.6%)	147 (47.1%)
Age at diagnosis (year)^a^	40.0 ± 13.5	39.2 ± 14.4	38.5 ± 12.4	39.5 ± 13.5
Age at PEG start (year)^a^	47.7 ± 13.4	45.1 ± 16.1	43.4 ± 13.5	46.2 ± 14.3
Age at inclusion in ACROSTUDY (year)^a^	49.4 ± 13.4	47.0 ± 16.0	45.2 ± 14.0	48.0 ± 14.4
Time between diagnosis and PEG start (year)^a^	7.8 ± 7.8	5.9 ± 6.0	4.9 ± 5.8	6.7 ± 7.1
Prior surgery				
No, *n* (%)	40 (24.0%)	25 (28.4%)	9 (15.8%)	74 (23.7%)
Yes, *n* (%)	127 (76.0%)	63 (71.6%)	48 (84.2%)	238 (76.3%)
Prior radiotherapy				
No, *n* (%)	119 (71.3%)	66 (75.0%)	44 (77.2%)	229 (73.4%)
Yes, *n* (%)	48 (28.7%)	22 (25.0%)	13 (22.8%)	83 (26.6%)
Prior SA				
No, *n* (%)	13 (7.8%)	3 (3.4%)	3 (5.3%)	19 (6.1%)
Yes, *n* (%)	154 (92.2%)	85 (96.6%)	54 (94.7%)	293 (93.9%)
Prior DA				
No, *n* (%)	100 (59.9%)	49 (55.7%)	37 (64.9%)	186 (59.6%)
Yes, *n* (%)	67 (40.1%)	39 (44.3%)	20 (35.1%)	126 (40.4%)
Concomitant SA				
No, *n* (%)	167 (100.0%)	0 (0.0%)	0 (0.0%)	167 (53.5%)
Yes, *n* (%)	0 (0.0%)	88 (100.0%)	57 (100.0%)	145 (46.5%)
Concomitant DA				
No, *n* (%)	136 (81.4%)	62 (70.5%)	39 (68.4%)	237 (76.0%)
Yes, *n* (%)	31 (18.6%)	26 (29.5%)	18 (31.6%)	75 (24.0%)

*PEG* pegvisomant, *GH* growth hormone, *DA* dopamine agonist, *SA* somatostatin analog

^a^Mean ± SD

In terms of previous therapy of the 312 patients enroled in this analysis, 238 (76.3%) had undergone surgery, 83 (26.6%) had undergone irradiation and 293 (93.9%) had been treated by SAs before inclusion in ACROSTUDY. There were no clinical differences between the three groups of patients in terms of past treatment history. The proportion of patients with concomitant treatment with DA was 24% (*n* = 75) in the whole cohort and 18.6% in the M group, 29.5% in the PB group and 31.6% in the SB group. The median follow-up duration was 80, 42 and 70 months in M, PB and SB groups, respectively.

### M vs. PB

For the first analysis, we compared the patient characteristics and treatment data between each treatment regimen at baseline (i.e., before PEG initiation) and after PEG initiation.

#### Comparison of baseline patient characteristics

**Fig. 1:**
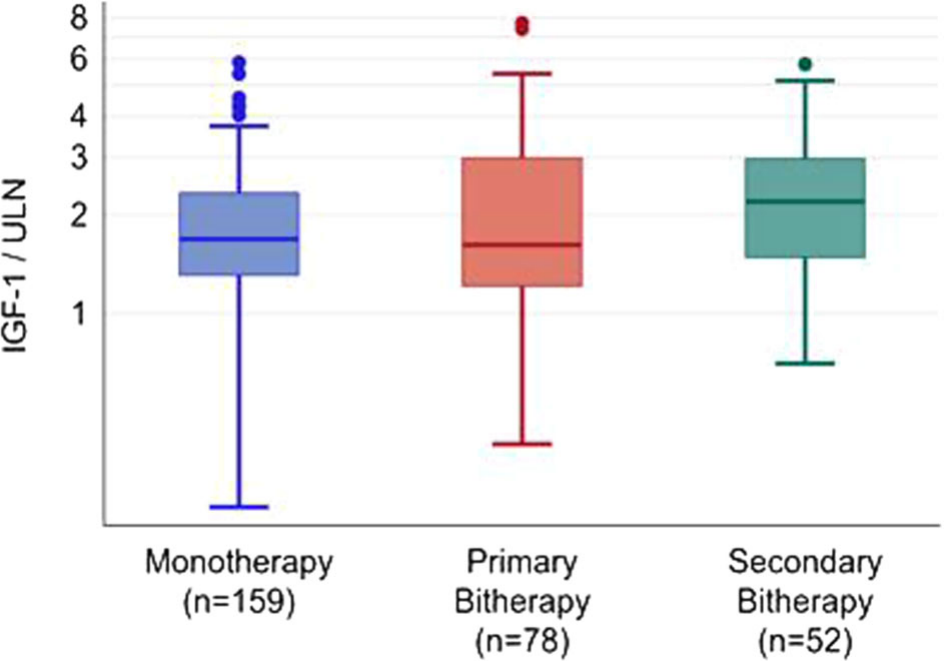
Baseline IGF-I levels (adjusted to the upper limit of normal IGF-I according to age and sex) in patients under monotherapy, primary bitherapy or secondary bitherapy

Before PEG therapy, the main patient baseline characteristics were as follows: geometric mean (Q1, Q3) IGF-I levels were similar in the M and PB groups [511.1 (386.0; 687.5) vs. 552.7 (356.5; 933.4) ng/mL, respectively, *P* = 0.291] (Fig. 1), as was the proportion of patients with supranormal age-adjusted IGF-I levels (89.9% vs. 84.6%, respectively, *P* = 0.302). Geometric mean GH levels (Q1, Q3) were also similar between both groups [2.94 (1.30; 6.70) vs. 4.25 (1.60; 10.40) ng/mL, respectively, *P* = 0.178)].

Regarding tumour status, the proportion of patients with suprasellar extension of the tumour was higher in the PB group than in the M group (67.5% vs. 44.4%, respectively, *P* = 0.022), while optic chiasm involvement was similar. Pituitary tumour shrinkage under SA prior to PEG initiation had been observed in a higher proportion of patients in the PB group than in the M group (36.5% vs. 17.8%; *P* = 0.012) while the proportion of patients who received previous operation or irradiation was similar in the M and PB groups (surgery, 76.0% and 71.6%, respectively, *P* = 0.438; radiotherapy, 28.7 and 25%, respectively, *P* = 0.524). Finally, the body mass index (BMI; 29.2 ± 6.4 kg/m^2^, in M patients vs. 28.0 ± 4.5, in PB patients, *P* = 0.265), the fasting glucose (5.89 ± 1.67 mmol/L in M patients vs. 5.68 ± 1.23 mmol/L, in PB patients, *P* = 0.439) and HbA1c (6.19 ± 1.03% in M patients vs. 6.32 ± 0.77% in PB patients, *P* = 0.511) were similar in both groups before PEG.

#### Comparison of patient characteristics under PEG combined to SA

**Fig. 2:**
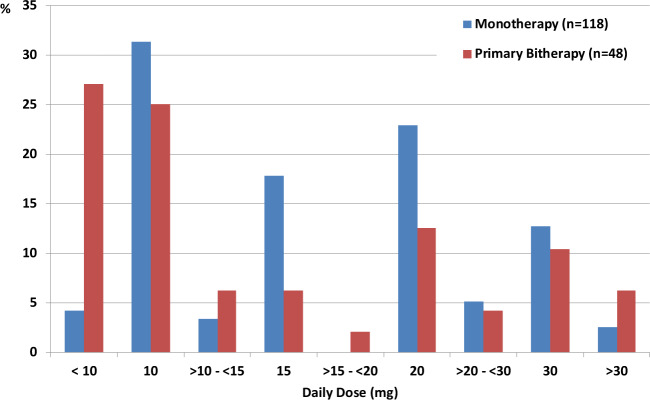
Proportion of patients treated with monotherapy or primary bitherapy who achieved normal IGF-I according to daily dose of pegvisomant (mg)

Under PEG, the aforementioned patient characteristics evolved as follows. The geometric mean (Q1, Q3) IGF-I level was lower in the M group than in the PB group [198.4 (151.8; 263.0) vs. 235.0 (152.0; 327.2) ng/mL, respectively, *P* = 0.024]. The proportion of patients with IGF-I levels below or equal to the upper limit of normal (ULN) tended to be higher in the M group than in the PB group (73.8% vs. 62.3%, *P* = 0.072). When considering IGF-I threshold levels ≤1.3 ULN and ≤2 ULN, the proportion of patients with such levels was significantly higher in the M group than in PB group (84.4% vs. 71.4%, respectively, *P* = 0.019 and 98.1% vs. 89.6%, respectively, *P* = 0.006). For patients who achieved normal IGF-I levels, the median daily dose was lower in the PB group than in the M group [10 (8.6; 20.0) vs. 15 (10.0; 20.0) mg/d, respectively, *P* = 0.036] (Fig. 2). Similar observations were identified in patients who achieved IGF-I < 1.3 ULN [10 (8.6; 20.0) vs. 15 (10.0; 20.0) mg/d, respectively, *P* = 0.027]. The median first PEG dose was similar in both groups (10 mg/day).

**Fig. 3:**
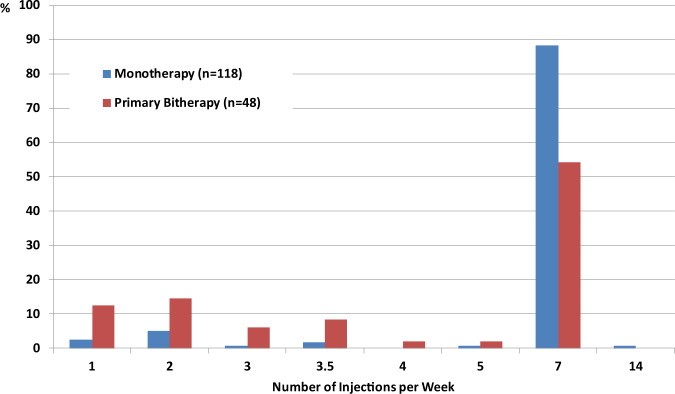
Number of pegvisomant injection per week in patients who achieved normal IGF-according to M or PB regimen

In terms of injection frequency, the number of injections per week was significantly lower in the PB group than in the M group, with 11.0% of patients in the M group and 45.8% of those in the PB group achieving normal IGF-I with less than seven injections per week (Fig. 3). This was also the case in patients with slightly (≤1.3 ULN) or moderately (≤2 ULN) elevated IGF-I (data not shown). During each year of follow-up, the mean weekly PEG dose was similar between both groups, with 103.3 vs. 99.4 mg at year 5 (group difference and 95% CI: −3.2 (−19.4; 13.6), *P* = 0.677).

**Fig. 4:**
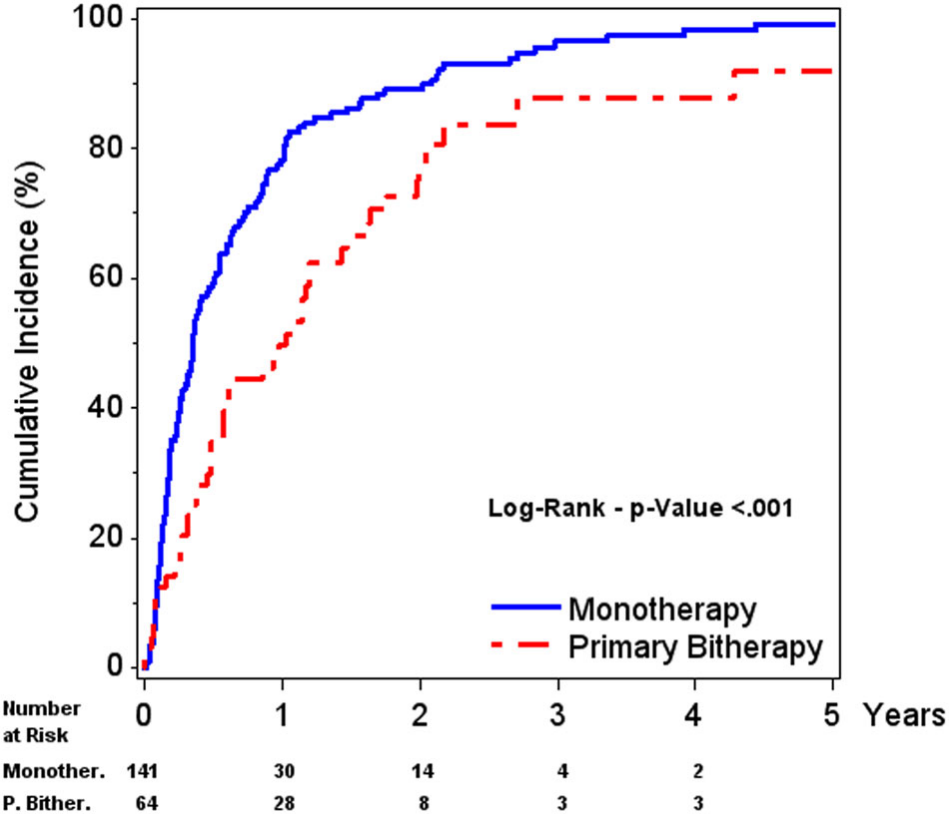
First IGF-I ≤ ULN - Cumulative incidence in patients under monotherapy or primary bitherapy (PEG added to SA). Population: patients with IGF-I > ULN at baseline and with at least one post-baseline IGF-I

Globally, the median time needed to achieve first normal IGF-I level was higher in the PB group than in the M group [7 (3.3; 14.4) vs. 4.2 (1.8; 10.3) months, respectively *P* = 0.019] (Fig. 4). For patients with IGF-I ≤ 1.3 ULN, the higher PEG dose that was used in the M group (median 15.0 mg), compared to that used in the PB group (median 10.0 mg), allowed a significantly higher normalisation rate (84.4% vs. 71.4%, respectively, *P* = 0.019).

In terms of safety, the same proportion of hepatic abnormalities was observed in each group: 11.5% and 9.8% in M and PB groups, respectively, for ALT > 2 ULN; 5.7 and 3.7% in M and PB groups, respectively, for AST > 2 ULN. The same trend was noticed for fasting glucose. Finally, an increased tumour size was observed in 14 of the 147 patients of the M group (9.5%) and in 10 patients of the 70 patients of the PB (14.3%) group (*p* = 0.296).

### M vs. SB

The SB regimen was considered for patients who were first treated PEG alone, after shifting from SA treatment in 54 patients out of 57 (94.7%). Patients were then administered a second treatment of combination SA + PEG. For this second analysis, we compared the characteristics and responses of patients during the three treatment periods: baseline (before PEG), during PEG and before SA, and after SA with PEG (SB).

#### Comparison of baseline patient characteristics

Before PEG initiation, the main patient baseline characteristics were as follows: 92.3% of patients in the SB group had IGF-I levels that were above normal, and a similar rate was observed for the M group (89.9%, *P* = 1.000). The basal geometric mean (Q1, Q3) IGF-I level was higher in the SB group than in the M group [642.5 (447.0; 870.0) vs. 511.1 (386.0; 687.5) ng/mL, respectively, *P* = 0.004] (Fig. 1). Geometric mean GH levels (Q1, Q3) were also similar between both groups [2.94 (1.30; 6.70) vs. 4.43 (2.10; 10.60) ng/mL, respectively, *P* = 0.212].

Regarding tumour status, the proportion of patients with suprasellar extension of the tumour was 50.0% in the SB group (*n* = 24) and 44.4% in the M group (*n* = 63), respectively, *P* = 0.642, while optic chiasm involvement was not different: 40.0% in the SB group (*n* = 25) and 31.3% in the M group (*n* = 44), respectively, *P* = 0.433. Pituitary tumour shrinkage under SA prior to PEG initiation had been observed in 19.4% of patients in the SB group (*n* = 36) and 17.8% in the M group (*n* = 90), respectively, *n* = *P* = 0.827).

#### Comparison of patient characteristics during PEG alone

During PEG and before starting SA, the geometric mean (Q1, Q3) IGF-I level was higher in the SB group than in the M group [344.0 (233.0; 489.6) vs. 198.4 (151.8; 263.0) ng/mL, respectively, *P* < 0.001], and only 33.3% of patients in the SB cohort had normalised IGF-I levels, compared to 73.8% of those in the M group (*P* < 0.001). Before starting SA, the median daily PEG dose was similar in the SB and M groups for patients with normalised IGF-I levels [15 mg (10.0; 20.0) respectively, *P* = 1.000], and for patients with IGF-I greater than ULN [20 mg (10.0; 30.0) vs. 22.5 mg (17.5; 30.0), respectively, *P* = 0.141]. The number of weekly injections was similar in patients with normal IGF-I of both groups.

The number of patients in whom an increase in tumour size was observed tended to be higher in the SB cohort (8 of 57 patients, 21.1%) than in the M cohort (14 of 167 patients, 9.5%) (*P* = 0.086). The proportion of patients with headaches did not significantly differ between groups (12.6% in M group and 8.8% in SB group, *P* = 0.439). There was also no difference in liver function abnormalities (ALT > 2 ULN occurred in 11.5% of patients of the M group vs. 11.6% of the SB group, AST > 2 ULN occurred in 5.7% of patients of the M group vs. 4.7% of the SB group) between groups.

#### Comparison of patient characteristics during PEG combined with SA

After SA addition to PEG, the proportion of patients in the SB group with normal IGF-I levels increased from 33.3 to 56.0%. Moreover, 16 of the 30 patients (53.3%) with IGF-I > ULN under PEG alone achieved normalised IGF-I levels after SA introduction (*P* = 0.003). The geometric mean (Q1, Q3) IGF-I level decreased under SB therapy (decrease = 34.3%, *P* < 0.001) but tended to remain higher in the SB group than in the M group [233.7 (159.6; 353.0) vs. 198.4 (151.8; 263.0) ng/ml, respectively, *P* = 0.060)]. The M group had a higher proportion of patients with normal IGF-I levels than the SB group (73.8% vs. 56.0%, *P* = 0.017). In the SB group, the median daily PEG dose did not change after SA introduction (20 mg/d before and after SA initiation), but the number of daily injections reduced for 27.3% of patients (*P* < 0.001). Of the seven analysable patients in the SB group in whom tumour size increased under PEG alone, SA addition stabilised the tumour volume in six, while the tumour continued to grow in the seventh patient. In three other patients whose tumour size did not change under PEG alone, an increased in tumour size was observed at the time of combination therapy.

Regarding safety, the same proportion of hepatic abnormalities was observed in each group: 11.5% and 8.5% in the M and SB groups, respectively, for ALT > 2 ULN (*P* = 0.567); 5.7 and 0.0% in M and SB groups, respectively, for AST > 2 ULN (*P* = 0.122). Lipohypertrophy was noticed in 1.2% and 0% of patients in the M and SB groups, respectively.

## Discussion

This retrospective and descriptive analysis showed that among patients who received PEG for acromegaly in France, PEG and SA combination was prescribed in almost half of them, confirming that the frequency of the combination is increasing [24]. We first analysed the reasons why, in some patients, physicians chose to prescribe PEG M (generally by switching patients from previous SA treatment to PEG), while in others, PEG was added to SAs (PB) or SAs were introduced after PEG M (SB).

For the PB group, a potential explanation for the choice of treatment regimen could be that the disease was more severe. In fact, the median IGF-I was similar in PB and M groups level before PEG initiation, as was the proportion of patients with normal age-adjusted IGF-I levels. Tumour mass could provide another explanation, particularly if previous SA treatment was able to reduce tumour mass or if any concerns about the optic chiasm were present. This explanation is plausible, as the proportion of patients with suprasellar extension was higher in the PB group than in the M group, even though optic chiasm involvement was similar between both groups. Moreover, pituitary tumour shrinkage under SA, prior to PEG initiation, was observed in a higher proportion of patients in the PB group. The potential for rebound from somatostatin-induced shrinkage is substantiated by reports that have shown that under PEG alone, the few somatotroph adenomas that grew were mainly those that previously shrunk under SA [25–27]. It is worth noting that from the global ACROSTUDY central MRI analysis, it was found that the occurrence of tumour volume increase/tumour progression was rare in patients under PEG [16]. On the contrary, a reason for preferring to switch to PEG alone (M group) rather than to continue SA with addition of PEG could be due to an increase in blood glucose levels with SA alone. Indeed, it has been shown that glucose tolerance improves when patients are treated by PEG [28–33]. This was probably not the case in our study, as BMI, fasting glucose and HbA1c levels were similar in both groups before starting PEG.

Regarding the SB group, our analysis revealed that the mean basal IGF-I level was higher in this group than in the M group, which suggests that IGF-I levels were more difficult to normalise in patients for whom SA was added to PEG, compared with patients who continued treatment with PEG alone. The fact that a higher proportion of patients in the SB group displayed tumour growth compared to those in the M group could also explain why physicians opted for addition of SA to PEG.

Another reason for combination therapy over PEG M is to reduce the frequency of injections [17, 20]. In the present analysis, 16% of patients of the M group received less than seven injections per week, compared to 44.3% of those in the PB group. If the first PEG dose for patients in the M and PB groups was similar (10 mg/day), the PB regimen allowed, in patients with well controlled IGF-I levels, a median daily PEG dose reduction. In contrast, the higher dose used in the M group, compared to the PB group, led to a higher rate of IGF-I normalisation, with a significantly lower time to achieve normality. Therefore, the potential explanations for initiating PB rather than PEG alone would appear to be due to concerns for tumour re-growth after SA interruption and/or the weekly number of injections, rather than the dose of PEG.

In the SB group, we did not observe any difference in terms of median daily PEG dose (20 mg), but the injection frequency decreased in a lower proportion of patients in the SB group than those in the PB group (27.3% of patients received less than seven injections per week). In the SB group, 53.3% of patients with supranormal IGF-I levels achieved IGF-I normalisation after SA addition to PEG. This confirms that the main reason for switching from M to bitherapy was due to partial control of the disease under PEG alone when a daily dose of 20 mg was reached. The benefit of this regimen is clear, as the proportion of patients with normalised IGF-1 levels increased at the same PEG dose (20 mg) as that used in the M group. However, it is important to note that the maximum dose recommended of PEG is 30 mg. An appropriate titration of PEG in M could also increase the proportion of patients with normalised IGF-1

According to an ACROSTUDY survey [24], another reason for switching from M to bitherapy was the presence of headaches. This was not confirmed in our study, where no differences in headache incidence was observed between M, PB and SB groups. Finally, in the same ACROSTUDY observational study [24], a tumour near the optic chiasm was a reason for switching to combination therapy. Likewise, in our SB group, an increased tumour size was observed after initiating PEG alone and explained secondary addition of SAs. Fortunately, it has been demonstrated that tumours shrink or at least stabilise under combination therapy [34], as we observed in some of our patients.

Finally, in the present analysis, the proportion of patients with IGF-I ≤ ULN under PEG M, under PEG combined with SA (PB) and SA added to PEG (SB) was 73.8%, 62.3% and 56.0%, respectively (Table 2). The efficacy of SA and PEG combination therapy has varied between studies, and IGF-I normalisation has been achieved in 58–90% of patients, depending on definition of efficacy and study design [16, 18, 19, 21, 23, 34–36]. Our results are in accordance with those observed in the 768 patients of the ACROSTUDY, where combination therapy resulted in a normalisation of IGF-I levels for 61% of the patients at 5 years [24].

**Table 2:** Normalisation and median dose by last IGF-I

	Monotherapy	PB	SB^a^
% patients with IGF-I < ULN	73.8%	62.3%	56%
Median dose (mg)—IGF-I < ULN^b^	15	10	20
% patients with IGF-I < 1.3 ULN	84.4%	71.4%	72%
Median dose (mg)—IGF-I < 1.3 ULN^c^	15	10	18.6

^a^SB: after SA addition to PEG

^b^Median dose for patients with IGF-I < ULN

^c^Median dose for patients with IGF-I < 1.3 ULN

This study confirms PEG treatment efficacy, as normalisation was achieved in 73.8% of patients in M group, 62.3% in PB and 56.0% in SB cohorts, at the respective median dose of 15, 15 and 20 mg. However, it is important to note that the baseline characteristics were different and that IGF-1 assays could vary between centres. The latter allowed IGF-I normalisation in nearly half of patients who did not achieve normalisation under PEG alone, which reinforces its application for patients with more severe disease. Nevertheless, our analysis of the three regimens shows that PEG dose titration may still not be optimal, especially in patients who maintain high IGF-I levels.

Furthermore, it should be noted for all three regimens, that the median daily PEG dose in patients whose IGF-I levels remained high never exceeded 20 mg, whereas the maximal dose recommended in the summary of product characteristics is 30 mg/day. It is interesting to note that the proportion of patients with increased IGF-I was similar in the patients treated with 30-mg PEG/day whatever their groups (M, PB and SB) (data not shown). Moreover, the fact that patients in the SB therapy group had higher IGF-I levels before any medical treatment certainly reflects a more severe disease and implies that they could benefit from higher PEG doses. These data reinforce the heterogeneous treatment responses of patients with acromegaly. The lack of optimal PEG dose titration has already been shown in a previous ACROSTUDY analysis [14], where IGF-I normalisation was more difficult for patients with higher baseline IGF-I levels, who were treated at a daily PEG M dose of 20 mg.

PEG treatment is well tolerated, and the most frequently reported side effects were lipodystrophy and hepatotoxicity. Lipohypertrophy refers to an increased subcutaneous fat deposition around the PEG injection site that is reversible by more frequent injection site rotations [37, 38]. Its prevalence, initially considered low (1.4%) in the ACROSTUDY [13], has been reported to be higher (15%) in a recent Spanish multicentre retrospective study [39]. The condition involves mostly females, and combined therapy may increase its incidence [40–42]. The variable prevalence may depend on how carefully the patients are inspected for this side effect. In our study, lipohypertrophy was noticed in 1.2%, 0% and 0% of patients in the M, PB and SB groups, respectively.

Hepatotoxicity could occur during PEG M or combination therapy, and this side effect is usually mild and transient [40–43]. In combination therapy, transient elevated transaminases, which were more than threefold greater than the ULN, were reported in 11.3–13.5% of patients [34, 43]. In our study the incidence of hepatic abnormalities (more than two times the ULN) was similar, whether patients had PEG alone or PEG with SAs, between primary or secondary biotherapy cohorts.

In conclusion, PEG and SA combination therapy is used in France through two different regimens. Some patients were also treated with DAs but in the similar proportion among our three groups. Our analysis showed that the medical rationale for primary combination, i.e., the co-prescription of PEG and SAs as soon as SAs alone do not control the disease, seems to depend on the concern for tumours with suprasellar extension and previous tumour shrinkage under SA. The reason for secondary combination therapy, i.e., the addition of SAs to PEG when the latter had been initially prescribed alone, involves the replacement of SAs when they fail to control acromegaly. This rationale appears clearer and applies to patients with increases in tumour size and IGF-I levels that do not normalise. In both regimens, the prospect of lowering the frequency of PEG injections favoured the choice, even though the mean weekly PEG dose between M and PB remained the same.

This study also confirms that personalised therapy with a combination of PEG and SAs may benefit patients who have tumours with growth potential. Consideration of patients’ quality of life can also guide the choice of treatments. The continuation of SAs when initiating PEG may also be justified for patients who have a more severe disease, as the reintroduction of SAs could improve patient outcomes when PEG is not sufficiently effective.
