# Correction to: Pegvisomant in combination or pegvisomant alone after failure of somatostatin analogs in acromegaly patients: an observational French ACROSTUDY cohort study

**DOI:** 10.1007/s12020-020-02532-w

**Published:** 2020-11-06

**Authors:** Emmanuelle Kuhn, Philippe Caron, Brigitte Delemer, Isabelle Raingeard, Hervé Lefebvre, Gérald Raverot, Christine Cortet-Rudelli, Rachel Desailloud, Clementine Geffroy, Robin Henocque, Yves Brault, Thierry Brue, Philippe Chanson

**Affiliations:** 1 Assistance Publique-Hôpitaux de Paris, Hôpital Bicêtre, Centre de Référence des Maladies Rares de l’Hypophyse HYPO, 94275 Le Kremlin-Bicêtre, France; 2 Université Paris-Saclay (Université Paris-Sud), Inserm, Signalisation Hormonale, Physiopathologie Endocrinienne et Métabolique, Le Kremlin-Bicêtre, France; 3 CHU de Toulouse, Hôpital Larrey, 24 Chemin de Pouvourville, TSA 30030, 31059 Toulouse Cedex 9, France; 4 CHU de Reims—Hôpital Robert Debré, Avenue du Général Koenig, 51092 Reims Cedex, France; 5 CHRU de Montpellier, Maladies Endocriniennes, Hopital Lapeyronie, 295 Avenue du Doyen Gaston Giraud, 34295 Montpellier Cedex 5, France; 6 CHU de Rouen, 1 Rue de Germont, 76031 Rouen Cedex, France; 7 Hospices civils de Lyon, Hôpital Louis Pradel, 59 Boulevard Pinel, 69677 Bron Cedex, France; 8 CHR Lille, Hôpital Claude Huriez, Rue Michel Polonovski, 59037 Lille, France; 9 CHU d’Amiens, Hôpital Nord, Place Victor Pauchet, 80054 Amiens Cedex 1, France; 10 Pfizer France, 23-25 Avenue du Docteur Lannelongue, 75668 Paris Cedex 14, France; 11 CHU de Marseille, Hôpital de la Conception, 147 boulevard Baille, 13385 Marseille Cedex 5, France

Correction to: Endocrine

10.1007/s12020-020-02501-3

The article Pegvisomant in combination or pegvisomant alone after failure of somatostatin analogs in acromegaly patients: an observational French ACROSTUDY cohort study, written by Philippe Chanson, was originally published electronically on the publisher’s internet portal (currently SpringerLink) on September 28, 2020 without open access. With the author(s)’ decision to opt for Open Choice the copyright of the article changed on November 2020 to © The Author(s) 2020 and the article is forthwith distributed under the terms of the Creative Commons Attribution 4.0 International License (http://creativecommons.org/licenses/by/4.0/), which permits use, duplication, adaptation, distribution and reproduction in any medium or format, as long as you give appropriate credit to the original author(s) and the source, provide a link to the Creative Commons license and indicate if changes were made.

The original article has been corrected.

Open Access This article is distributed under the terms of the Creative Commons Attribution 4.0 International License (http://creativecommons.org/licenses/by/4.0/), which permits unrestricted use, distribution, and reproduction in any medium, provided you give appropriate credit to the original author(s) and the source, provide a link to the Creative Commons license, and indicate if changes were made.

